# From Academia to Reality Check: A Theoretical Framework on the Use of Chemometric in Food Sciences

**DOI:** 10.3390/foods8050164

**Published:** 2019-05-14

**Authors:** Vi Khanh Truong, Madeleine Dupont, Aaron Elbourne, Sheeana Gangadoo, Piumie Rajapaksha Pathirannahalage, Samuel Cheeseman, James Chapman, Daniel Cozzolino

**Affiliations:** 1 Nanobiotechnology Laboratory, School of Science, College of Science, Engineering and Health, RMIT University, Melbourne, VIC 3001, Australia; vi.khanh.truong@rmit.edu.au (V.K.T.); madeleine.dupont@rmit.edu.au (M.D.); aaron.elbourne@rmit.edu.au (A.E.); sheeana.gangadoo@rmit.edu.au (S.G.); s3758115@student.rmit.edu.au (P.R.P.); s3741431@student.rmit.edu.au (S.C.); james.chapman@rmit.edu.au (J.C.); 2 Food Science and Technology, Bundoora Campus, School of Science, College of Science, Engineering and Health, RMIT University, Melbourne, VIC 3086, Australia

**Keywords:** chemometrics, food, quality, analysis

## Abstract

There is no doubt that the current knowledge in chemistry, biochemistry, biology, and mathematics have led to advances in our understanding about food and food systems. However, the so-called reductionist approach has dominated food research, hindering new developments and innovation in the field. In the last three decades, food science has moved into the digital and technological era, inducing several challenges resulting from the use of modern instrumental techniques, computing and algorithms incorporated to the exploration, mining, and description of data derived from this complexity. In this environment, food scientists need to be mindful of the issues (advantages and disadvantages) involved in the routine applications of chemometrics. The objective of this opinion paper is to give an overview of the key issues associated with the implementation of chemometrics in food research and development. Please note that specifics about the different methodologies and techniques are beyond the scope of this review.

## 1. Introduction

Advances in biology, biochemistry, chemistry, and mathematics have increased our knowledge and understanding of the main issues facing food systems (e.g., food integrity, safety, omics) [1,2,3]. Nevertheless, only a limited number of interactions and cause–effect associations reported in foods are well understood, due to the intricate nature of these relationships, which has hindered our ability for a fundamental knowledge of these singularities in food and consequently the development of new innovations to boost R&D and new applications in the food industry [1,2,3].

The so-called bottom-up approach *(“reductionist”)* has dominated research in food science, where only one compound or nutrient was considered or analyzed independent of the food matrix [4]. It is in this context that several scholars in the field have detailed that this line of thinking has created an “*unreal world view*”, where chemical components/molecules/nutrients (e.g., protein, carbohydrates, lipids) analyzed in isolation from the whole food matrix (e.g., beer, fruits, flour, grains) might not be solely responsible for explaining the observed differences in the food [5,6,7,8,9,10]. In recent years, the understanding of the inherent complexity in food will require intricate answers, hence, studies in this space must move in the direction of a more holistic, multidisciplinary, and integrative arrangement (e.g., systematic approach) as stated by several researchers in the field [5,6,7,8,9,10].

**Figure 1:**
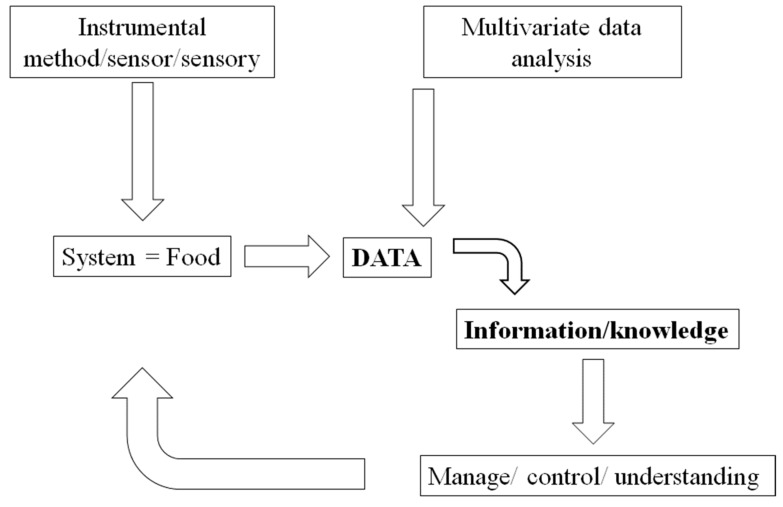
A schematic representation of the application of multivariate data analysis in the food industry.

This new approach (e.g., systems, omics) to the analysis of food and food systems will have the capacity to determine a high level of complexity which has not been explored before by food scientists where for many researchers in the field is still considered a “scientific utopia” [5,6,7,8,9,10]. In the last 30 years, the modern food industry (and sciences) has moved into the digital and technological age, providing better tools able to deal with the numerous challenges which result from the use of novel instrumental techniques and methods, hardware (e.g., computing, mobile telecommunications) and software (algorithms). The combination of these methods and techniques have been incorporated into the exploration, mining, and description of the data from such complexity (see Figure 1). Nevertheless, in this digital environment, food scientists need to be mindful of the issues (advantages and disadvantages) involved in the routine application of modern analytical and instrumental methods during the analysis of food and food systems [5,11,12,13,14,15].

**Figure 2:**
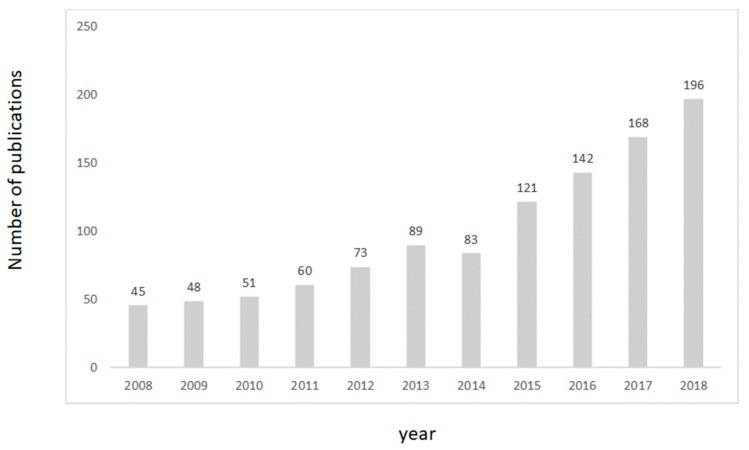
Number of citations including the words chemometric, multivariate data analysis, and food (source Web of Science).

Encompassing this approach, researchers in food science have been proactive in the integration and evaluation of multivariate data analysis methods (chemometrics), as demonstrated by the increased number of published articles in the field (over 1300 articles have been published containing keywords such as “food”, “chemometrics”, and/or “multivariate data”) (Web of Science, March 2019). During the last decade, more than 1000 papers alone were published demonstrating and reporting the ability of these methods to target issues related with food integrity and safety, authenticity, and applications of instrumental methods and techniques (e.g., near infrared, mid infrared, Raman, electronic noses and tongues), to mention a few examples. This exponential growth in the number of available articles can be explained by the accessibility to instruments and chemometric software by both researchers and industry, allowing for the development of new applications. Please note that the keywords used in this search only included the words chemometrics, multivariate data analysis, and food (see Figure 2). 

The objective of this opinion paper is to give an overview of the key issues associated with the implementation of chemometrics in food research and development. Please note that specifics about the different methodologies and techniques are beyond the scope of this review.

## 2. Chemometrics Linking the Univariate with the Multivariate World

According to Hopke (2003) [5] the assimilation of modern statistical and chemometric methods and techniques in food R&D have become more important since the 1980s. Unfortunately, the universal application of chemometrics in food science has been endangered by the selection or inappropriate use of the different techniques or methods available as highlighted by Nunez and co-workers (2015) [2]. These authors also emphasized that several of these studies ignored the basic requirements for a proper experimental design before incorporating these methodologies into the analysis of food [2,3,16]. Furthermore, it has been shown that among the main steps required in the effective application of such statistical methods in food sciences (research and development) were the non-interest in performing complex mathematical analysis which prevailed among scientists and determined the misinterpretation of the statistical results or the misuse of the statistical packages available to perform the analysis [2,3,16]. Contradictorily, the ease of access to a wide range of commercial statistical software (some of them available in modern instrumental techniques) have provided researchers with valuable tools that facilitated the incorporation of statistical and mathematical methods to explore and develop new applications [2,3,16].

The development and fast growth in the use of technology (e.g., computing power) has had an important influence on the routine applications of mathematics and analytics in food science (e.g., R&D) due to the availability of computing packages, facilitating the statistical analysis of different types of datasets [2,3,16]. This has granted researchers the potential of analyzing data from food experiments in a smaller amount of time to generate models, charts or even resolve complex mathematical processes by using diverse types of algorithms and pre-processing techniques. The availability of these tools has also facilitated the rapid expansion and integration of these methodologies in food sciences; however, this has also resulted in the inappropriate use of such methods where there is a necessity of both understanding the mathematics that stands behind the model as well as the digital tools needed to create models/charts, etc. [2,3,16]. In many cases, misleading or wrong conclusions were drawn based on the lack of understanding of the chemometric background. Granato and collaborators [3] (2014) highlighted on a positive note, the importance of the integration and use of these approaches in a new era in food research and development.

Tremendous gains have been made in analytical capabilities resulting from the integration of a diverse array of methods and techniques in food science. Despite this, researchers still face several issues; topics associated with the practicality of the information gained from the data generated (e.g., need for data integration tools), the requirements for rigorous control systems in order to verify the integrity of the data generated, among other factors, are still some constraints that face the user of these methods in food sciences [5,11,12,13,14,15].

A recent review defined chemometrics as “the application of statistical and mathematical methods, to handle chemical or process data” [17]. This review also highlighted that a more comprehensive definition of chemometrics was introduced by Massart and colleagues [18], where according to this scientists chemometrics includes mathematics, statistics, and formal logics to design and/or decide on the optimal experimental design, as well as to maximize the comprehensive interpretation of chemical information from chemical data, to gather deep knowledge about the system [17,18,19]. It is in this space that the widespread application of chemometrics in the modern food sciences is concerned with issues related to the analysis of big and multivariate data [17,18,19]. 

The routine use of statistics in food R&D focuses on the investigation of the effects of single variables (e.g., univariate) by means of standard statistical analysis (e.g., analysis of variance). Although, the routine use of ANOVA in research delivers valuable data, detailed information about associations among variables as well as other relevant information related with sampling, the sample or the experiment might be missing [8,9,20,21].

Nowadays, large datasets (e.g., several variables and samples) are collected each day in most of the labs and industrial sites around the world due to the introduction of modern instrumental methods [8,9,20,21,22]. Additionally, the standard and conventional methods and techniques currently in use lean towards the elimination of the matrix interference detaching or removing the analyte (e.g., chemical or physical) that is measured, determining an apparent simple analytical process [8,9,20,21,22,23]. Nevertheless, this systematic method allows for a better understanding of the intrinsic associations between the several constituents and properties that define a food.

The main characteristic of the various rapid analytical and instrumental techniques used by the food industry is that, in most cases, the parameters estimated (e.g., measured) during the analysis, do not necessarily have a direct link with the analyte of interest, resulting in a proxy or correlative method [8,9,20,21,22,23,24,25,26]. Alternatively, chemometric methods offer to analyze food beyond the one-dimensional (univariate) space. Therefore, the use of chemometric analysis and interpretation of the data can reveal properties, relationships, and levels of interferences or interactions in the food matrix not easily observed when univariate analysis is used [8,9,20,21,22,23,24,25,26]. 

Researchers are concerned about the quantifiable interactions between dependent and independent variables to gain evidence and data about the system (e.g., interactions, models, simulation charts, among others). In quantitative analysis, the development of a linear function or model that connects dependent and independent variables is one of the common applications of these methods in food R&D [24,27,28,29]. Regression and calibration might be interchanged and used as a single word in reporting calibrations (e.g., fit a model) or to quantify the associations between variables [24,27,28,29]. However, it is important to remember that this association does not necessarily determine a cause–effect relationship [24,27,28,29]. Table 1 summarizes the most common algorithms used in several of the applications of chemometrics in food R&D.

## 3. The Importance of Experimental Design

Before sample analysis, data collection, mining, and interpretation of the results, the design (e.g., treatments, variables) of the experiment (DoE) is considered of fundamental importance in this approach [32,33,34]. However, this significant first step is usually overlooked or misjudged in many of the applications or analysis reported. Moreover, the DoE founded in the assumption that only one variable change relative to others, is no longer valid when “state-of-the-art” instrumental techniques and chemometrics are combined for the analysis of complex systems such as food [32,33]. Recent applications of chemometric methods and techniques highlighted as prerequisite the need for optimization of the variables in combination with the appropriate DoE protocol to carry out the experiments [32,33]. It has been demonstrated that good DoE not only provides with the means of exploring different and several factors or interactions at the same time but also provides with an efficient tool to make savings in routine applications of any given method [32,33,34].

## 4. Sampling and Samples

The most often misjudged component of any analysis, which has a vital part during model building and mining of the data generated, are the sampling process and the sample itself [35,36,37]. One of best-known applications of chemometric methods in the food industry is the development of calibration models [35,36,37]. During this process, finding a “robust model” encompasses, among other issues, the cautious collection of appropriate samples to be incorporated into the model (e.g., calibration development) [35,36,37].

The sampling method and the selection of the sample are undoubtedly the most important stages to be consider before developing a calibration model [35,36,37]. This process involves different stages, which ideally results in the selection of a wide range of samples covering current and future sources of variability (e.g., range in protein, temperature, moisture) to be subsequently measured [35,36,37]. Samples in both the training and test must belong to the same population (e.g., origin, similar chemical or physical properties) as the model will not be able to predict samples outside these settings [35,36,37].

Consequently, samples collected with the purpose of being included in calibration must hold the different and expected levels of variability (training and validation) where selected samples must be equally distributed throughout calibration and validation [35,36,37]. Any further sample to be incorporated into the model would be exposed to identical conditions (e.g., temperature, moisture, treatments, etc.) to those in the training set. The purpose of this is to generate the broadest range in composition to compensate for unwanted variations in the system, during the test and routine use of the model [35,36,37].

## 5. Interpretation of Results and Validation

In most applications of instrumental methods, the main objective is the creation of a model or calibration to predict unknowns [30,38,39,40,41,42]. However, before the calibration model is used in the real world, it must be validated [30,38,39,40,41,42]. The validation process requires the model to predict the desired property using several samples not involved during the calibration process [40]. Any results obtained after the validation must be compared with the reference value; if both values are identical (the exception of the rule), the model can be used to accurately predict the property in the future [30,38,39,40,41,42]. 

Based on the data available in the published reports, cross validation has been the favorite tool to check the capability of the model to predict new samples [30,38,39,40,41,42]. However, in some cases such as in the so called “*bottom-up approach*” the exploratory research starts with the mining of well-known analyses. In this scenario, a given scientist experienced in these new methodologies compares results through interlaboratory studies. It is in these cases that cross-validation is of great utility.

However, the capability of the model to accurately predict the unknowns must be tested using a proper dataset (e.g., test or validation sets) [30,38,39,40,41,42]. In practice, to test the predictive ability of the calibration model, an independent set of samples must be used (e.g., samples not used when developing the calibration) [30,38,39,40,41,42]. The general rule of thumb is that independent samples should be sourced from other experiments, batches or conditions (e.g., harvests, different temperatures, moisture content, origins) [30,38,39,40,41,42,43,44]. A common strategy followed by many researchers is to split the dataset into two subsets, the calibration (used to construct the model) and the validation set (used to test the model) using different methods or algorithms such as principal component analysis (PCA), Bayesian, Kennard–Stone algorithms, and/or random sampling [30,38,39,40,41,42,43,44]. All these methods have positive or negative implications when used to select or design the most appropriate sample to be incorporated into the model [30,38,39,40,41,42,43,44].

As reported by other authors, numerous statistics and acronyms have been described to interpret the results obtained during calibration development and validation experiments [30,38,39,40,41,42,43,44]. These statistics include the prediction error of a calibration model, which is defined as the root mean square error for cross validation (RMSECV), when cross validation is used, or the root mean square error for prediction (RMSEP) when internal or external validation is used [30,38,39,40,41,42,43,44]. These statistics provide an estimation of the average uncertainty that can be expected for predictions of future samples [30,38,39,40,41,42,43,44]. The standard error of prediction (SEP) can be reported instead of the RMSEP [30,38,39,40,41,42,43,44]. The residual predictive deviation (RPD) value has also been proposed to evaluate the ability of a calibration model to predict new samples [44,45]. The RPD value is defined as the ratio of the standard deviation of the response variable to the RMSEP or RMSECV (other authors use the term SDR) [44,45].

A common statistic often used to report the capability of the model to predict new samples is the coefficient of determination (R^2^). This statistic represents the proportion of explained variance of the response variable in either the training or test sets [28,45]. Overall, the relationships between variables can be defined by the existence of some structured association (linear, quadratic, etc.) between the independent (X) and dependent (Y) variables [28,45]. It is generally accepted that correlation quantify how strong the association between two variables is where the robustness of the prediction is usually linked with the ability of the model to measure or predict the future behavior or results that it is designed to predict [28,45]. 

Unfortunately, different authors have described and reported similar results using different statistics/acronyms, making the assessment and interpretation of results published in the literature very difficult. One of the most important issues is related to the differences in the magnitude and structure of the population with respect to the measured parameter (e.g., range, standard deviation, coefficient of variation). It is therefore critical to report the standard deviation (SD), minimum and maximum values of the population for the attribute of interest [28,45]. 

Most applications report or interpret the models using the statistical parameters described above. However, this is not sufficient to describe the model and other parameters such as the loadings or coefficients of regression need to be added into the interpretation or reporting of the results (e.g., the why and how of the analysis). Even though the model is established, the fit for purpose criterion needs to be included during the evaluation of the models. Therefore, the users of these technologies need to interpret the models in the overall context of the application and not only on the cold interpretation of the statistics [46].

Another important step during calibration and validation which contributes towards the robustness of the models is the incorporation of appropriate pre-processing methods [21,22,23,24,30,38]. This step is of importance when instrumental methods are used (e.g., GC, HPLC) during analysis as the chromatogram needs some degree of pre-processing before being used for data mining (e.g., peak alignment, standardization). Numerous pre-processing methods have been proposed by different authors such as the use of derivatives (e.g., first and second), smoothing, bias, and slope corrections [21,22,23,24,30,38]. A detailed description and explanation of these pre-processing methods is beyond the scope of this report and the reader is directed to consult relevant references on the topic. 

## 6. The Misuse of Chemometrics

The development of new applications (e.g., sensory, instrumental and advance methods) in food science either in research or by the industry have been boosted using chemometrics [47,48]. However, a word of caution on potential biased practice of this method in food science is needed [47,48]. Several issues must be considered during the development of quantitative models such as aspects about the sample selection (e.g., number, replicates, origin) critical to develop the models, the need of independent validation (not just cross validation), as well as the appropriate selection of pre-processing, among other issues, are still the most common errors made by the practitioners of chemometrics [3,33,47,48].

Of importance before embracing the use of chemometric methods is the definition of the exact purpose of the analysis as food “quality” can be defined as fitness for purpose. Most of the reports in the use of chemometrics highlighted the importance of defining the fitness for purpose as this can be associated with aspects of sampling (e.g., origin of the sample, the number of samples) and the process analyzed. Central to the development and utilization of these methods is the need to understand that the results are only as good as the sampling method and the DoE. Numerous examples indicated that calibration and models will be invalid due to inadequacies in sampling (e.g., sample selection). 

Finally, after the model or calibration was established, the validation and systematic update of the models must be considered and implemented. It is important to remember that the overall error of the model developed (e.g., model error) will be of the accumulation of errors (squared) during all the steps followed during the process (e.g., DoE, sampling, laboratory errors, and mistakes, etc.) [3,33,47,48]. Table 2 summarizes drawbacks and missuses of the application of chemometrics gathered from published reports.

## 7. Final Considerations and Perspectives 

The growing uptake of rapid analytical methods and techniques in food science, in either research or industry, was boosted by the increasing use of mathematics and statistics (e.g., software, algorithms, internet of things, databases) as they become a key component of the analysis. While the development of such applications in food science could be considered a simple computational exercise, the whole process needed to be considered. To this end, researchers must develop a comprehensive understanding of the complexity of the analysis (system) where the integration of the sample into the evaluation and mining of the data, the role of the instrument (e.g., signal-to-noise ratio), the soundness of the multivariate data analysis method selected, and the end use are components of the analytical system.

Modelling in modern food science relies on gathering data to generate knowledge, as well understanding different aspects of the system that might influence the analysis of samples or processes. Among the most important factors that govern the incorporation of chemometric techniques into the analysis and the interpretation of the results are (i) knowledge about the reference laboratory method (reported as the standard error of the laboratory), (ii) intrinsic characteristics of the method or technique selected (e.g., limit of detection, extraction steps, etc.), (iii) inherent characteristics and properties of the sample (e.g., chemical and physical properties), and (iv) associations between the sample, instrument and the data collected (e.g., signal-to-noise ratio, peak alignment, drifts, pre-processing). Different to the routine use of analytical methods, this approach will also require that the person in charge of developing such models needs to have the knowledge about the whole process used to generate the sample, and the willingness to engage in multidisciplinary work. 

The current developments and uses of chemometrics in food science are related to the prediction of nutritional value and functional properties evaluated with instrumental methods. The ability to simultaneously evaluate multiple parameters in a single analysis has revolutionized the way that instrumental methods are used, allowing for the development of new applications. Future progress of these developments will provide analytical tools to interrogate about the composition or variations in commodities and foods in real time in addition to testing the integrity of the foods (undesirables or faults, food safety, traceability, and origin, among others).

Nevertheless, the inadequate academic support in topics such as novel use of instrumental techniques and chemometrics, are among the several limitations which faces the routine utilization of these methods by both the food industry and R&D. Even though there are some academic organizations that actively interact with the food industry sharing knowledge and expertise enriching their experience with mutual benefits for both parties. Regrettably, these are still several roadblocks for the widespread of these methods in food R&D and their translation into the food industry. 

## Figures and Tables

**Table 1:** Commonly used algorithms and corresponding application in food research and development.

Algorithm	Acronym	Application/s	Reference
Multiple Linear Regression	MLR	Calibration/modelling/prediction	[20,21,22,23,24,25,30]
Partial Least Squares	PLS		
Principal Component Regression	PCR		
Discriminant Analysis	DA	Classification	[20,21,22,23,24,25]
Cluster Analysis	CA	Classification	[20,21,22,23,24,25]
Partial Least Squares-Discriminant Analysis	PLS-DA	Classification	[20,21,22,23,24,25,30]
Linear Discriminant Analysis	LDA	Classification	[20,21,22,23,24,25,30]
Soft Independent Modelling of Class Analogies	SIMCA	Classification based in PCA	[20,21,22,23,24,25,30]
Principal Component Analysis	PCA	Outlier detection	[31]
		Data visualization/inspection	
		Revealing relationships (e.g., between variables, between samples)	
		Finding patterns	

**Table 2:** Summary of common drawbacks and mistakes encountered during the application of chemometrics in food science, research, and development.

Common Drawbacks and Mistakes
Lack of understanding of the chemometric tools (e.g., background, limitations of the method)
Diverse type of algorithms and pre-processing techniques (e.g., improper selection of the appropriate tool for the task)
Lack of the fundamentals and information required to interpret the results
Incorrect use or sampling protocol
Lack or inappropriate experimental design
Inappropriate sample selection (e.g., number of samples, source)
Validation (e.g., cross-validation versus independent validation)
Issues reporting results (e.g., no information about the laboratory error associated with the reference method; Inconsistencies in reporting errors)
Lack or minimal training/education
Easy access to hardware and software

## References

[1] Fotakis C., Kokkotou K., Zoumpoulakis P., Zervou M. (2013). NMR metabolite fingerprinting in grape derived products: An overview. Food Res. Int..

[2] Nunes C.A., Alvarenga V.O., Sant’Ana A.D.S., Santos J.S., Granato D. (2015). The use of statistical software in food science and technology: Advantages, limitations and misuses. Food Res. Int..

[3] Granato D., Calado V.M.D.A., Jarvis B. (2014). Observations on the use of statistical methods in Food Science and Technology. Food Res. Int..

[4] Fardet A. (2014). New Approaches to Studying the Potential Health Benefits of Cereals: From Reductionism to Holism. Cereal Foods World.

[5] Hopke P.K. (2003). The evolution of chemometrics. Anal. Chimi. Acta.

[6] Burlingame B. (2004). Holistic and reductionist nutrition. J. Food Compos. Anal..

[7] Cozzolino D., Daniel C. (2015). Foodomics and infrared spectroscopy: from compounds to functionality. Curr. Opin. Food Sci..

[8] Munck L. (2007). A new holistic exploratory approach to Systems Biology by Near Infrared Spectroscopy evaluated by chemometrics and data inspection. J. Chemom..

[9] Munck L., Møller J.B., Rinnan Å., Fast S.H., Møller E.M., Nørgaard L., Balling E.S. (2010). A physiochemical theory on the applicability of soft mathematical models—Experimentally interpreted. J. Chem..

[10] Kelly J.G., Trevisan J., Scott A.D., Carmichael P.L., Pollock H.M., Martin-Hirsch P.L., Martin F.L. (2011). Biospectroscopy to metabolically profile biomolecular structure: A multistage approach linking computational analysis with biomarkers. J. Proteome Res..

[11] Capozzi F., Bordoni A. (2013). Foodomics: A new comprehensive approach to food and nutrition. Genes Nutr..

[12] Granato D., Putnik P., Bursać Kovačević D., Sousa Santos J., Calado V., Silva Rocha R., Gomes Da Cruz A., Jarvis B., Rodionova O.Y., Pomerantsev A. (2018). Trends in Chemometrics: Food Authentication, Microbiology, and Effects of Processing. Compr. Rev. Food Sci. Food Saf..

[13] Castro-Puyana M., Mendiola J.A., Ibáñez E. (2013). Strategies for a cleaner new scientific discipline of green foodomics. TrAC Trends Anal. Chem..

[14] Cevallos-Cevallos J.M., Reyes-De-Corcuera J.I., Etxeberria E., Danyluk M.D., Rodrick G.E. (2009). Metabolomic analysis in food science: A review. Trends Food Sci. Technol..

[15] Wishart D.S. (2008). Metabolomics: Applications to food science and nutrition research. Trends Food Sci. Technol..

[16] Buco S.M. (1990). How good are your results? An approach to qualitative and quantitative statistical analysis for food monitoring and process control systems. Food Control.

[17] Matero S., vanDen Berg F., Poutiainen S., Rantanen J., Pajander J. (2013). Towards Better Process Understanding: Chemometrics and Multivariate Measurements in Manufacturing of Solid Dosage Forms. J. Pharm. Sci..

[18] Massart D.L., Vandegiste B.G.M., Deming S.N., Michotte Y., Kaufman L. (1988). Chemometrics: A Textbook.

[19] Mutihac L., Mutihac R. (2008). Mining in chemometrics. Anal. Chim. Acta.

[20] Wold S. (1995). Chemometrics; what do we mean with it, and what do we want from it?. Chemom. Intell. Lab. Syst..

[21] Martens H.M.A.M. (2001). Multivariate Analysis of Quality. An Introduction. Meas. Sci. Technol..

[22] Adams M.J., Barnett N.W. (1995). Chemometrics in analytical spectroscopy. RSC Spectroscopy Monographs.

[23] Otto M. (1999). Chemometrics: Statistics and Computer Application in Analytical Chemistry.

[24] Esbensen K.H. (2002). Multivariate Data Analysis in Practice.

[25] Geladi P. (2003). Chemometrics in spectroscopy. Part I. Classical chemometrics. Spectrochim. Acta Part B.

[26] Woodcock T., Downey G., O’Donnell C.P., Downey G. (2008). Better Quality Food and Beverages: The Role of near Infrared Spectroscopy. J. Near Infared Spectrosc..

[27] Møller S.F., Von Frese J., Bro R. (2005). Robust methods for multivariate data analysis. J. Chemom..

[28] Asuero A.G., Sayago A., Gonzalez A.G. (2006). The Correlation Coefficient: An Overview. Crit. Rev. Anal. Chem..

[29] Brereton R.G. (2008). Applied Chemometrics for Scientist.

[30] Naes T., Isaksson T., Fearn T., Davies T. (2002). A User-Friendly Guide to Multivariate Calibration and Classification.

[31] Bro R., Smilde A.K. (2014). Principal component analysis. Anal. Met..

[32] Leardi R. (2009). Experimental design in chemistry: A tutorial. Anal. Chim. Acta.

[33] Granato D., Calado V.M.A., Granato D., Ares G. (2014). The use of importance of design of experiments (DOE) in process modelling in food science and technology. Mathematical and Statistical Approaches in Food Science and Technology.

[34] Szczepanska N., Kudłak B., Namiesnik J. (2018). Recent advances in assessing xenobiotics migrating from packaging material e A review. Anal. Chim. Acta.

[35] Murray I. (1999). NIR spectroscopy of food: simple things, subtle things and spectra. NIR News.

[36] Murray I., Cowe I., Roberts C.A., Workman J., Reeves J.B. (2004). Sample preparation. Near Infrared Spectroscopy in Agriculture.

[37] Cozzolino D. (2014). Sample presentation, sources of error and future perspectives on the application of vibrational spectroscopy in the wine industry. J. Sci. Food Agric..

[38] Nicolai B.M., Beullens K., Bobelyn E., Peirs A., Saeys W., Theron K.I., Lammertyn J. (2007). Non-destructive measurement of fruit and vegetable quality by means of NIR spectroscopy: A review. Post Harvest Biol. Tech..

[39] Walsh K.B., Kawano S., Zude M. (2009). Near infrared spectroscopy. Optical Monitoring of Fresh and Processed Agricultural Crops.

[40] Westad F., Marini F. (2015). Validation of chemometric models—A tutorial. Anal. Chim. Acta.

[41] Cozzolino D., Cynkar W.U., Dambergs R.G., Shah N., Smith P. (2009). Multivariate methods in grape and wine analysis. Int. J. Wine Res..

[42] Cozzolino D., Shah N., Cynkar W., Smith P. (2011). A practical overview of multivariate data analysis applied to spectroscopy. Food Res. Int..

[43] Norris K.H., Ritchie G.E. (2008). Assuring specificity for a multivariate near-infrared (NIR) calibration: The example of the Chambersburg Shoot-out 2002 data set. J. Pharm. Biomed. Anal..

[44] Fearn T. (2002). Assessing calibrations: SEP, RPD, RER and R2. NIR news.

[45] Williams P.C., Williams P.C., Norris K.H. (2011). Implementation of Near-Infrared technology. Near Infrared Technology in the Agricultural and Food Industries.

[46] Williams P., Dardenne P., Flinn P. (2017). Tutorial: Items to be included in a report on a near infrared spectroscopy project. J. Near Infrared Spectrosc..

[47] Badertscher M., Pretsch E. (2006). Bad results from good data. Trends Anal. Chem..

[48] Kjeldahl K., Bro R. (2010). Some common misunderstandings in chemometrics. J. Chemom..

